# Antisense phosphorodiamidate morpholino oligomers retain activity in *Burkholderia cepacia* complex biofilm

**DOI:** 10.3389/fmicb.2025.1660799

**Published:** 2025-09-23

**Authors:** Antonio R. Mendez, Christine Pybus, David E. Greenberg

**Affiliations:** ^1^Department of Internal Medicine, Infectious Diseases & Geographic Medicine, University of Texas Southwestern Medical Center, Dallas, TX, United States; ^2^Department of Biological Sciences, University of Texas at Dallas, Richardson, TX, United States; ^3^Department of Microbiology, University of Texas Southwestern Medical Center, Dallas, TX, United States

**Keywords:** antisense–oligonucleotide-drug conjugates, PPMOs, *Burkholderia cepacia* complex (Bcc), biofilm, antibiotic development

## Abstract

**Background:**

Members of the *Burkholderia cepacia* complex (Bcc) are known to cause severe pulmonary infections in immunocompromised hosts, namely individuals with cystic fibrosis (CF) and chronic granulomatous disease (CGD). Due to innate antibiotic-resistant phenotypes and the formation of protective biofilms, Bcc bacteria are difficult to eradicate from colonized lungs using traditional antibiotics. An alternative therapeutic approach involves the use of antisense molecules, specifically peptide-conjugated phosphorodiamidate morpholino oligomers (PPMOs). Previously, we found that PPMOs targeting the acyl carrier protein (AcpP) can reduce the burden of planktonic Bcc bacteria.

**Methods:**

Antimicrobial activities of AcpP PPMOs were assessed against established biofilms produced by Bcc clinical isolates, in which viable cells, biomass, and metabolic activity were enumerated. Bactericidal effects were further evaluated by microscopy. Cytotoxicity of these molecules was tested in human pulmonary cell lines.

**Results:**

AcpP PPMO treatment resulted in over three-log reductions (*p* < 0.0001) in biofilm burden across five clinical isolates of Bcc that were tested. A dose-dependent effect was observed (5–40 μΜ). This effect was visualized using confocal and scanning electron microscopy. We further demonstrated that PPMOs associate with bacterial cells in a time-dependent fashion using a fluorescently labeled AcpP PPMO with *B. cenocepacia* K56-2 DsRed. Finally, alveolar cells retained viability with AcpP PPMOs at bactericidal dosages.

**Conclusion:**

The Bcc biofilm setting is not a deterrent against PPMO delivery or antimicrobial activity. This is supported by the colocalization of AcpP PPMOs with cells, membrane destruction, loss of cell viability, and biomass reduction. Collectively, these data provide evidence that AcpP PPMOs are a promising therapeutic strategy in the treatment of Bcc infections.

## Introduction

The *Burkholderia cepacia* complex (Bcc) encompasses a group of Gram-negative, non-spore-forming bacilli. While phenotypically similar, these bacteria are genetically distinct and currently include at least 20 unique species that inhabit diverse ecological niches (Martina et al., 2018; Ong et al., 2016; Furuya et al., 2000; Compant et al., 2008; Kikuchi et al., 2005). However, among its members, the majority of human disease is attributed to *B. cenocepacia* and *B. multivorans* (Reik et al., 2005). These bacteria are opportunistic pathogens capable of causing severe pulmonary infections in certain hosts, such as individuals with cystic fibrosis (CF) and chronic granulomatous disease (CGD) (Lipuma, 2005; Greenberg et al., 2009). Further, nosocomial infections have emerged due to Bcc contamination in antiseptic agents and pharmaceutical products (Tavares et al., 2020).

The Bcc affects a minority of patients with CF (3–4%); however, these pathogens are capable of patient-to-patient transmission and inflict a broad range of disease burden, from asymptomatic infection to rapidly progressive, fatal pneumonia with systemic dissemination (Cepacia syndrome) (Isles et al., 1984; Mahenthiralingam et al., 2005; Rubin, 2018). Epidemic strains, such as those derived from the ET-12 lineage, have been a particular cause of concern due to their increased transmissibility and association with outbreaks (Jones et al., 2004; Chen et al., 2001). In these infections, traditional antibiotic approaches have limited efficacy due to the intrinsic antimicrobial resistance exhibited by the Bcc (Mahenthiralingam et al., 2005; Aaron et al., 2000; Abbott and Peleg, 2015; Sass et al., 2011). Furthermore, the Bcc readily forms robust biofilms *in vitro*, a trait that has been shown to augment drug resistance (Messiaen et al., 2014; Conway et al., 2002; Caraher et al., 2007; Desai et al., 1998). This trait has also been observed in chronic lung infections (Hansen et al., 2010; Hoiby et al., 2017; Lee et al., 2017) and is thought to contribute to recalcitrant infections by aiding host immune cell evasion, decreasing susceptibility to small-molecule antibiotics *in vivo*, and facilitating co-colonization with *Pseudomonas aeruginosa* (a common CF pathogen) (Mahenthiralingam et al., 2005; Tomlin et al., 2001; Riedel et al., 2001; Murphy and Caraher, 2015). Consequently, new antimicrobials are urgently needed to treat infections caused by these resistant organisms.

**Figure 1:**
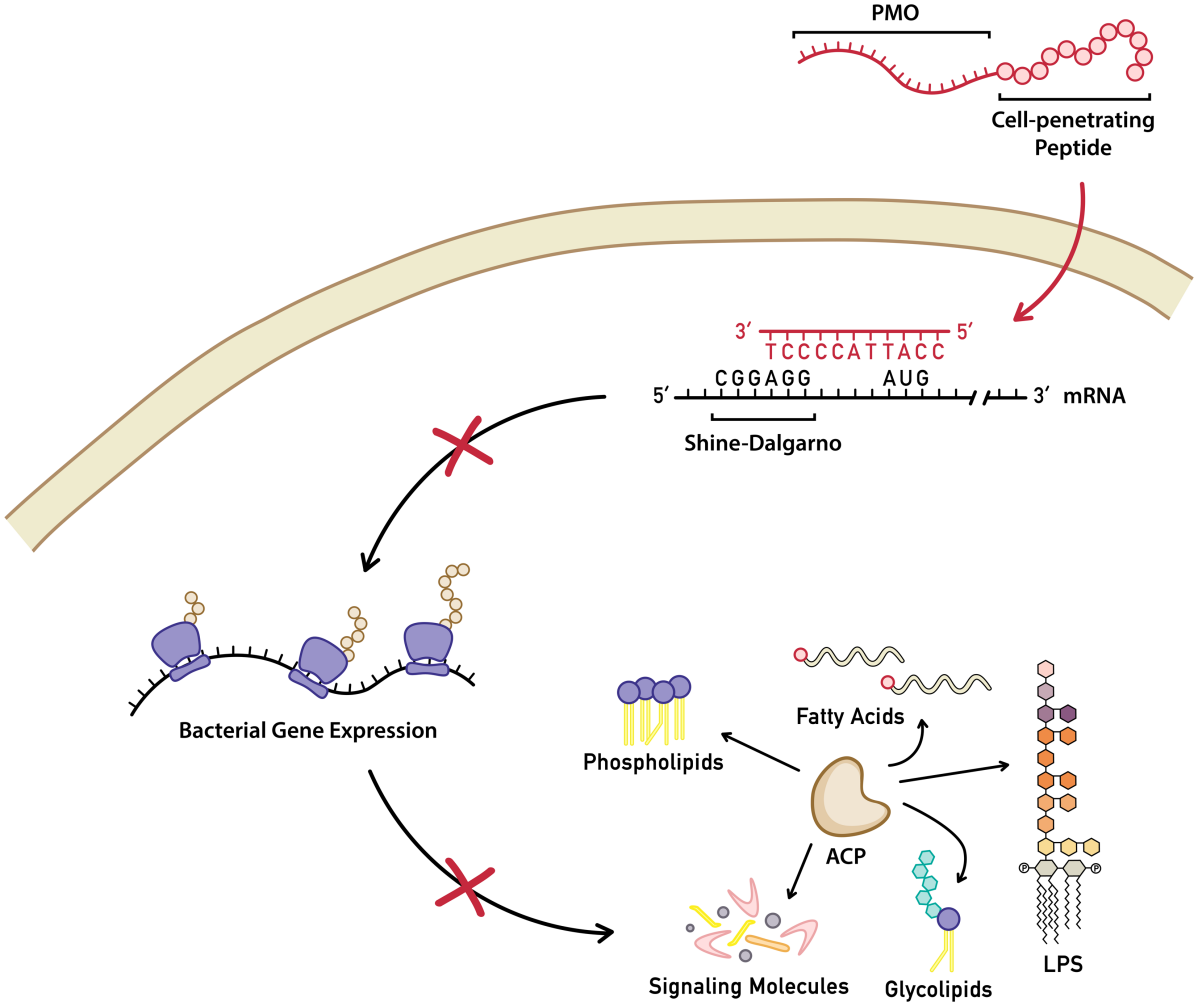
Structure and proposed mechanism of action of PPMOs. Inside a bacterial cell, peptide-conjugated phosphorodiamidate morpholino oligomers (PPMOs) targeting the *acpP* gene bind to mRNA near the Shine–Dalgarno sequence, sterically inhibiting translation. This inhibition disrupts the essential functions of the acyl carrier protein.

One proposed solution is the use of antisense molecules, specifically phosphorodiamidate morpholino oligomers (PMOs). These are synthetic oligonucleotides (11 bases long) designed to bind near the Shine–Dalgarno sequence and the translational start site of their gene target, thereby inhibiting its translation (Figure 1). The PMO contains a morpholino ring and a phosphorodiamidate linkage in place of a phosphodiester bond, which confers resistance to nuclease degradation. Additionally, PMOs are conjugated to a peptide for enhanced bacterial membrane penetration (peptide-conjugated PMO or PPMO) (Pifer and Greenberg, 2020).

Previously, PPMOs directed against a *Burkholderia* gene involved in fatty acid synthesis, *acpP* (acyl carrier protein), demonstrated bactericidal activity *in vitro* and *in vivo* (Greenberg et al., 2010; Daly et al., 2018). However, it remains unclear whether these PPMOs retain their activity against *Burkholderia* in the biofilm setting. Here, we report that AcpP PPMOs effectively eradicate the biofilm burden of five Bcc clinical isolates. We also demonstrated that fluorescently labeled AcpP PPMO associates with Bcc cells within a biofilm. Our findings indicate that the Bcc remains susceptible to antisense molecules, despite the protection provided by the biofilm microenvironment. In addition, we performed scanning electron microscopy (SEM) to characterize PPMO activity and performed *in vitro* toxicity assays in the human cell line A549 (type II pneumocyte).

## Methods

### Media and growth conditions

*Burkholderia* strains were isolation streaked from 25% glycerol/Mueller–Hinton Broth II (MHII) frozen stocks onto 5% sheep blood plates (Remel, Lenexa, KS) and incubated at 37 °C with 5% CO_2_. For minimum biofilm eradication concentration (MBEC) assays, isolated colonies were inoculated into 5 mL MHII and incubated overnight at 37 °C with shaking (220 rpm). The cultures were resuspended in 2 mL Dulbecco’s phosphate-buffered saline (DPBS, Gibco, Grand Island, NY) to an optical density (O. D.) of 600 nm at 0.08 (~1 × 10^8^ CFUs/mL). This suspension was then diluted in MHII to obtain a concentration of 5 × 10^5^ CFU/mL, which was used to inoculate an MBEC plate (Innovotech, Edmonton, Alberta, Canada).

### Bacterial strains

The bacterial strains used in this study are described in Table 1. A plasmid with a DsRed marker, pIN63, was a gift from Dr. Annette Vergunst (Université de Montpellier, Nîmes, France) (Vergunst et al., 2010).

**Table 1:** Minimum inhibitory concentration (MIC) values of peptide-conjugated phosphorodiamidate oligomers (PPMOs) for *Burkholderia* strains used in this study.

Strain	Species	Source	Minimum inhibitory concentration (μM)		
			AcpP-0077	AcpP-0791	Scr-0394
25416	*B. cepacia*	Onion (ATCC Stock)	16	16	>64
AU0062	*B. cenocepacia*	CF patient	4	8	>64
AU0158	*B. dolosa*	CF patient	4	16	>64
K56-2	*B. cenocepacia*	CF patient (ET12 lineage)	16	8	>64
SH-2	*B. multivorans*	CGD patient (Lung biopsy)	4	4	>64
HI4277	*B. cenocepacia*	CF patient (ET12 lineage)	32	16	>64

### Peptide-conjugated phosphorodiamidate oligomers (PPMO)

**Figure 2:**
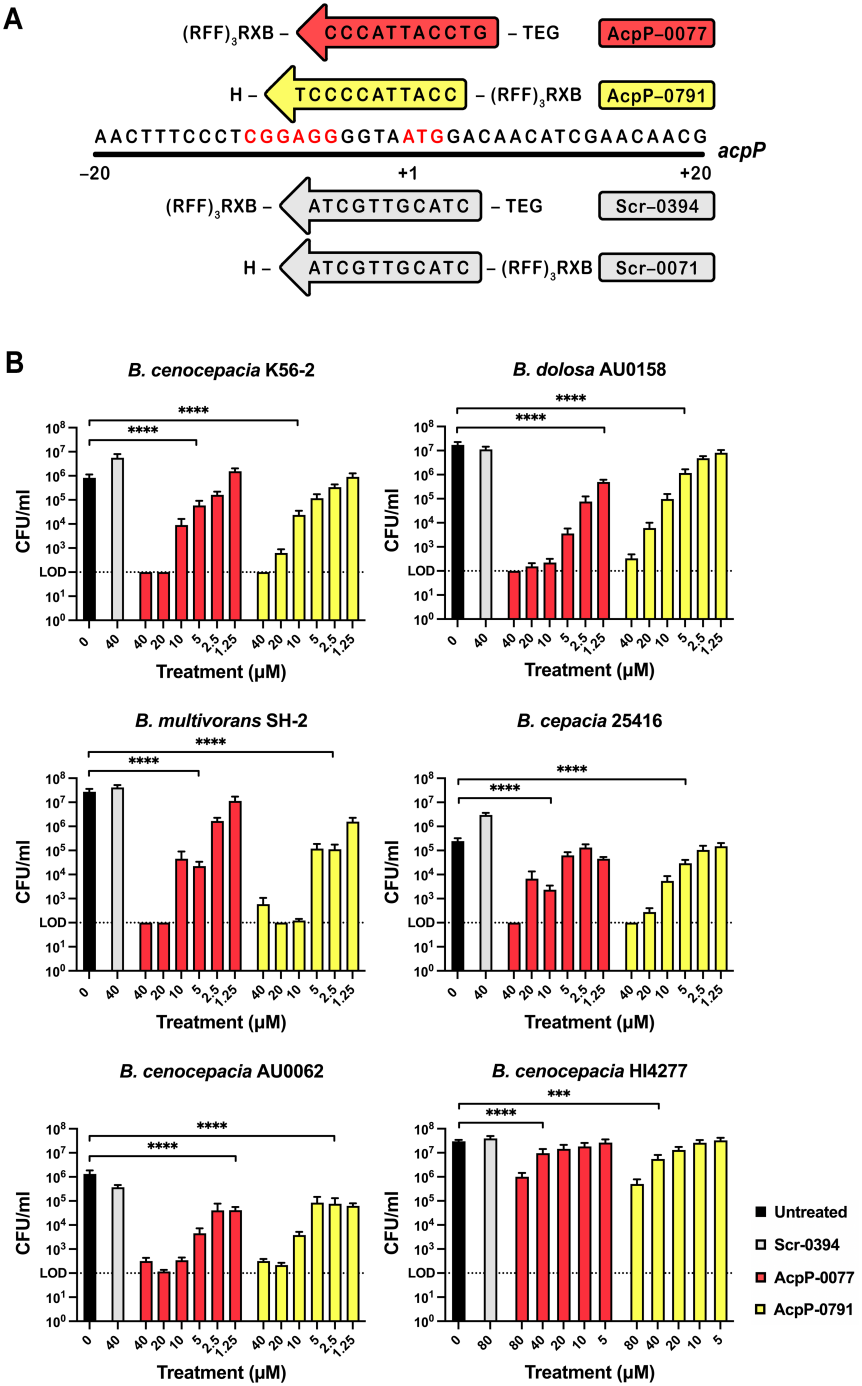
AcpP PPMOs retained bactericidal activity in the biofilms of clinical Bcc strains. **(A)** AcpP peptide-conjugated phosphorodiamidate morpholino oligomers (PPMOs) aligned with the *acpP* gene sequence of *B. cenocepacia* K56-2, with their conjugated peptides at the indicated 5′ or 3′ attachment site (X indicates 6-aminohexanoic acid, B indicates *β*-alanine). Scr–0394 and Scr-0071 PPMOs (gray) are included for comparison. The Shine–Dalgarno sequence and ATG start site are highlighted in red. **(B)** Biofilms were grown on MBEC pegs in MHII for 24 h, then treated with serially diluted doses of PPMOs at 24, 32, and 40 h. After a total of 48 h, the biofilms were quantified by mean (±SEM) viability counts reported as CFU/mL. Significance of biofilm eradication compared to the untreated control is indicated as ***p < 0.001 or ****p < 0.0001. The data represent at least three independent experiments, each with three technical replicates, and were analyzed using two-way ANOVA with Dunnett’s multiple comparisons test. The dashed lines represent the limit of detection.

Sarepta Therapeutics synthesized PPMOs (Cambridge, MA), as previously described (Figure 2A) (Howard et al., 2017; Tilley et al., 2006).

### Minimum inhibitory concentration (MIC) assays

The minimum inhibitory concentration (MIC) of each PPMO was assayed for all strains used in this study, as previously described (Table 1) (Daly et al., 2018; Howard et al., 2017).

### Biofilm reduction assays

A bacterial suspension of 150 μL in MHII was inoculated into each well of an MBEC plate. The plate was sealed with Breathe-Easy membrane (MidSci, St. Louis, MO) and incubated for 24 h at 37 °C with shaking (110 rpm). The lid with inoculator pegs was then placed in a 96-well plate containing fresh MHII with AcpP PPMO, a scrambled control PPMO, or antibiotics (UTSW Campus Pharmacy) serially diluted from a starting concentration. The plate was sealed and incubated as described above. This treatment was repeated every 8 h for 24 h (3 treatments in total). The pegs were then processed for viability assays, resazurin reduction assays, or crystal violet staining.

To measure the reduction of biofilm viability, the inoculator pegs were washed in DPBS and processed for colony-forming units as follows: each peg was cut at the base and placed into a sterile tube containing 1 mL of DPBS. The tubes were sonicated in a water bath for 15 min, vortexed, and serially diluted. Viable cells were enumerated on sheep blood agar by drip plating. For the resazurin assay, the lid with inoculator pegs was washed in DPBS and placed into a fresh 96-well plate containing 150 μL of MHII with 100 μM resazurin (Sigma-Aldrich, St. Louis, MO). After incubating for 3–6 h at 37 °C, fluorescence at Ex/Em 530/590 nm was measured using a Cytation 5 imaging reader (Biotek Instruments Inc., Winooski, VT). The crystal violet assay was processed as previously described (Howard et al., 2017).

For daily dosing experiments, a biofilm was grown in an MBEC plate and assayed for viability as described above. However, the samples were treated once a day with MHII containing either a PPMO or a scrambled control PPMO for 1–7 days. All biofilm assays included an untreated control and were repeated at least 3 times, with 3 technical replicates for each condition.

### Scanning electron microscopy

SEM analysis was performed on the biofilm formed by *B. multivorans* SH-2. An MBEC plate was prepared and treated like the biofilm reduction assay, using an AcpP PPMO and a scrambled control PPMO. An untreated control was included. MBEC pegs were rinsed in DPBS, cut using a hot scalpel, and then fixed in 2.5% (v/v) glutaraldehyde/0.1 M sodium cacodylate buffer overnight at 4 °C. The samples were processed as previously described (Munaweera et al., 2018), with the addition of 3 final washes in 100% ethanol during dehydration. Air-dried specimens were mounted on SEM stubs and sputter-coated with gold palladium for 1–2 min (Cressington 108 auto sputter coater). Images were acquired using a field emission scanning electron microscope (Zeiss Sigma) in high-vacuum mode at an accelerating voltage of 3.5 kV at the UT Southwestern Electron Microscopy Core Facility.

### MBEC assay and laser scanning confocal microscopy for the localization of fluorescently labeled PPMO within the biofilm

A culture of *B. cenocepacia* K56-2 carrying pIN63 (DsRed plasmid) was resuspended to 5 × 10^5^ CFU/mL in MHII. Into each well of a 96-well glass plate, 150 μL of this suspension was inoculated in triplicate. The plate was incubated for 72 h, with the medium replaced every 24 h. After 72 h, the medium was replaced with MHII containing 8 or 16 μM fluorescein-labeled AcpP PPMO-0791 or with MHII alone as a no-treatment control. A fluorescein-only control and an unlabeled cell control were also included. The plate was incubated at 37 °C for 1, 3, and 5 h. At each time point, the medium was removed from a set of wells, washed with DPBS, and fixed with 2.5% glutaraldehyde. The plate was imaged using a Zeiss 780 Airyscan laser scanning confocal microscope with a 63X/NA 1.4 oil immersion lens. Red channel excitation was 561 nm with the emission bandpass set at 570–616 nm. Green channel excitation was 488 nm with the emission bandpass set at 490–527 nm. All channels were imaged sequentially. Linear unmixing was used to account for autofluorescence in the green channel.

### Image analysis

The images were deconvoluted using Autoquant X3 software (Media Cybernetics, Inc., Rockville, MD). Volumetrically rendered images were constructed with deconvoluted files in Imaris software (Bitplane USA, Concord, MA). The images were analyzed using Imaris software to assess the degree of colocalization between the PPMO and bacteria. The bacteria were masked as the region of interest (ROI) before thresholding and analysis. The thresholded Pearson’s coefficient of colocalization within the ROI was calculated to assess the overlap between the red channel (bacteria expressing DsRed) and the green channel (fluorescein-labeled PPMO). At least three fields of view were examined, and representative images are shown.

### Cell viability assay

A549 alveolar epithelial cells (ATCC) were maintained in Dulbecco’s Modified Eagle’s Medium (DMEM) with 10% fetal bovine serum, glutamine, penicillin–streptomycin, and phenol red (DMEM medium) at 37 °C with 5% CO_2_. A tissue culture-treated 96-well plate was seeded with 0.1 mL per well of 4 × 10^4^ A549 cells/mL in the DMEM/F12K medium (with glutamine, no phenol red or antibiotics). After incubating for 48 h, the cells were washed with 0.1 mL DPBS, and 0.1 mL of the DMEM clear medium (no phenol red or penicillin–streptomycin) was added. Then, the cells were treated in triplicate with AcpP PPMO, scrambled sequence control PPMO, or vehicle (water) for 24 h. A fixed volume of 2 μL was used to obtain a final PPMO concentration of 5–80 μM. The cells were incubated for 24 h, and LDH release was quantified using the CyQUANT LDH Cytotoxicity Assay (Invitrogen, Eugene, OR). After calculating cytotoxicity following the manufacturer’s protocol, relative viability was determined (100% × 1 – cytotoxicity). The assay was repeated 3 times, with each repetition conducted in triplicate.

### Statistical analysis

The level of biofilm reduction for each AcpP PPMO, relative to the untreated control group, was assessed by two-way ANOVA of the geometric means, followed by Dunnett’s multiple comparisons test, using GraphPad Prism 9 software (GraphPad Software, Boston, MA).

## Results

### PPMOs reduced the established biofilm burden

A variety of *Burkholderia* species, including clinical, epidemic, and environmental strains (Table 1), were used to evaluate the activity of AcpP PPMOs in the biofilm setting. AcpP-0077 and AcpP-0791 (Figure 2A) were selected because each spans the Shine–Dalgarno sequence and ATG site, features shown to increase the potency of PPMOs in the Bcc (Daly et al., 2018).

First, the MICs of AcpP-0077 and AcpP-0791 were determined for these strains and found to be 4–32 μM in the planktonic setting (Table 1). Then, Bcc strains were grown for 24 h in MBEC plates to develop biofilms. The established biofilms were treated at 24, 32, and 40 h post-inoculation with 1.25–40 μM of AcpP-0077, Acp-0791, or Scr-0394 (scrambled sequence control)(*B. cenocepacia* HI4277 was tested at 5–80 μM). At 48 h, biofilm reduction was calculated by enumerating viable cells (Figure 2B). In our survey, five of the six Bcc strains challenged with either AcpP PPMOs demonstrated >3-log reductions (*p* < 0.0001) in the biofilm burden compared to the untreated controls, and treatment responses were dose-dependent (5–40 μM). The scrambled sequence controls were comparable to the untreated controls across all strains.

Eradication of the established biofilms was further assessed by quantifying metabolic activity and total biofilm mass. The resazurin assay measures metabolic activity using a blue resazurin dye that is reduced to pink resorufin in the presence of NADPH. The AcpP PPMO-treated MBEC biofilms showed a significant reduction in metabolic activity for all Bcc strains, except *B. cenocepacia* HI4277 (previously BCC1616), which displayed a more modest reduction (Supplementary Figure S1). Several MBEC biofilms were also analyzed using the crystal violet assay (Supplementary Figure S2), which measures total biofilm burden, including exopolymeric substances and live and dead bacteria. Both AcpP-0077 and AcpP-0791 reduced the *B. dolosa* AU0158 burden to less than 50% of the untreated control. Interestingly, the scrambled sequence control PPMO also affected the burden for unknown reasons. AcpP PPMOs also effectively reduced *B. cenocepacia* K56-2 biofilm, although the effect of AcpP-0077 was more potent than AcpP-0791. The total burden was less reduced in *B. cenocepacia* AU0062.

**Figure 3:**
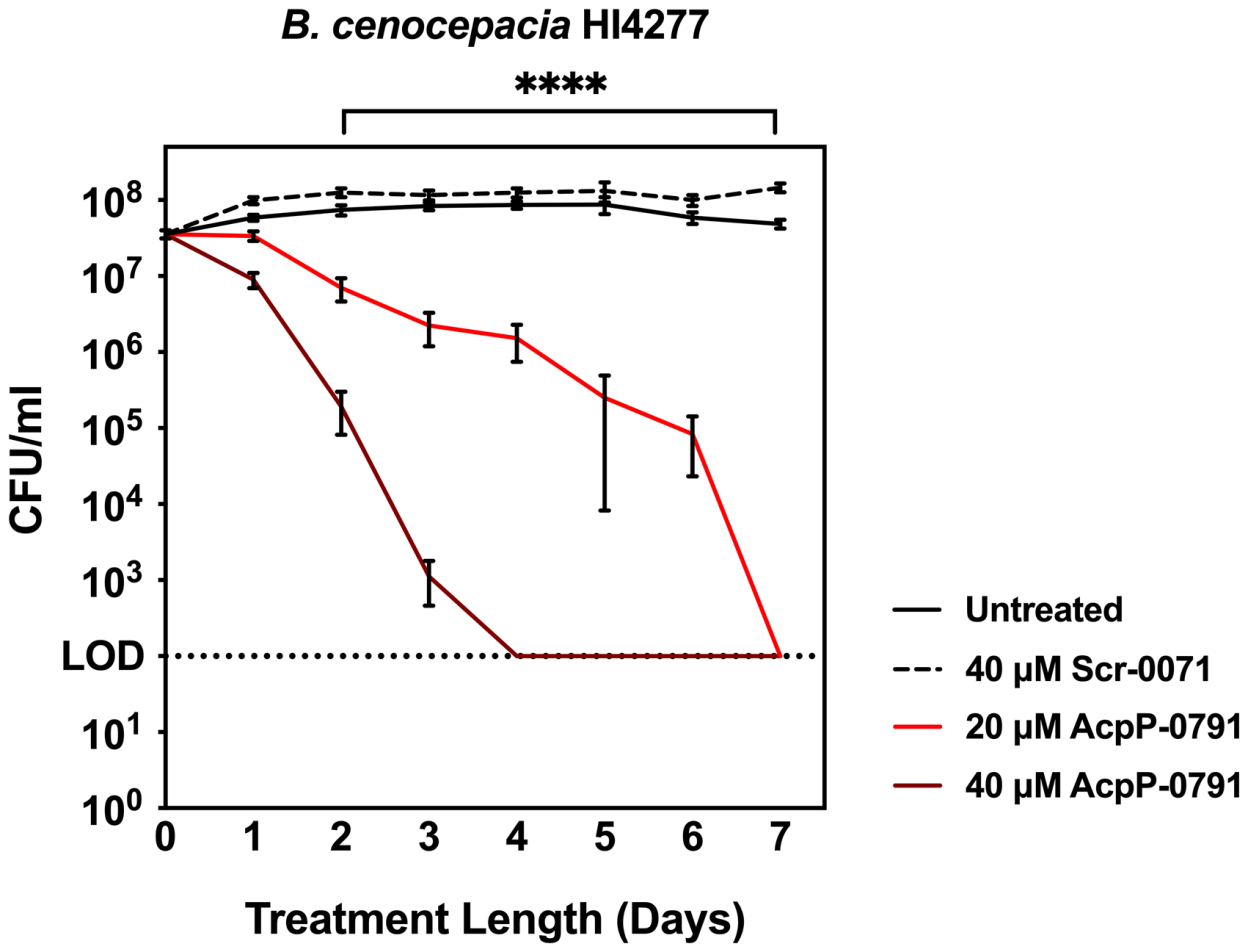
Daily dosing with AcpP PPMOs demonstrated bactericidal activity against the *B. cenocepacia* HI4277 biofilm. Biofilms were grown, quantified, and analyzed as described in Figure 2. Samples were treated daily for 1–7 days with 20 μM Scr-0071, 20 μM AcpP-0791, or 40 μM AcpP-0791. An untreated control was included. Significance of biofilm eradication in the AcpP-0791 treatment groups compared to the untreated control is indicated as ****p < 0.0001. The data represent three independent experiments, with three technical replicates each. The limit of detection is indicated by the dashed line.

We noted that the epidemic (ET-12 lineage) strain *B. cenocepacia* HI4277 exhibited decreased susceptibility to the PPMOs in the viability assay (Figure 2B). The maximum treatment response was observed at 80 μM, yielding 1.8- and 1.5-log reductions in viable cells (*p* < 0.0001) for AcpP-0791 and AcpP-0077, respectively. Treatment with 40 μM doses resulted in a proportionately halved dose–response (*p* < 0.001). Indeed, resistance was also observed when this strain was treated with two commonly used classes of antibiotics, including the *β*-lactams meropenem (MIC 64 μg/mL) and ceftazidime (MIC 128 μg/mL), as well as sulfamethoxazole/trimethoprim (MIC 64 μg/mL) (Supplementary Figure S3). This high resistance to PPMOs and small-molecule antibiotics is in line with prior observations, which have also reported a comparatively slower growing phenotype (Sass et al., 2011; Daly et al., 2018). We then assessed an alternative dosing schedule, in which a 24-h biofilm was treated with AcpP-0791 daily, over the course of 7 days (Figure 3). This strategy yielded >5.5-log reductions (*p* < 0.0001) in viable cells by day 4 (40 μM) and day 7 (20 μM). The scrambled sequence group remained analogous to the untreated control.

### AcpP PPMOs disrupted the bacterial cell membrane

**Figure 4:**
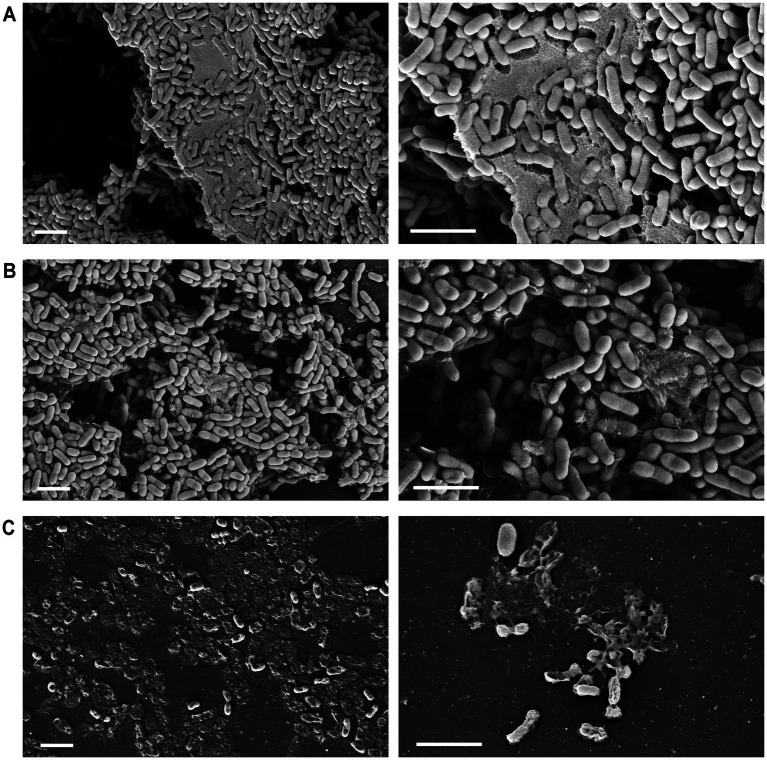
Scanning electron microscopy of PPMO-treated bacteria. *B. multivorans* SH-2 biofilms were grown for 24 h in an MBEC plate and then treated with PPMOs at 24, 32, and 40 h. After a total of 48 h, pegs with the treated biofilms were fixed, processed, mounted, and then examined using SEM. Illustrated is **(A)** the untreated biofilm compared to the biofilms treated with **(B)** 40 μM Scr-0394 or **(C)** 40 μM AcpP-0077 PPMO at magnifications of 5 K X (left) and 10 K X (right). Scale bars represent 2 μM of distance.

The bactericidal effects of the AcpP PPMOs were further evaluated using scanning electron microscopy (SEM). In the same manner as the MBEC assay, *B. multivorans* SH-2 was grown in an MBEC plate and subsequently treated. After fixing and processing, the samples were examined using SEM, which revealed large aggregations of cells with abundant extrapolymeric substances (EPS) present in both the untreated and scrambled sequence control groups (Figures 4A,B). However, in the 40 μM AcpP-0077 group, visualization showed widespread cell membrane lysis, cell debris, and remnants of EPS (Figure 4C).

### AcpP PPMOs associated with *Burkholderia* in a biofilm matrix

**Figure 5:**
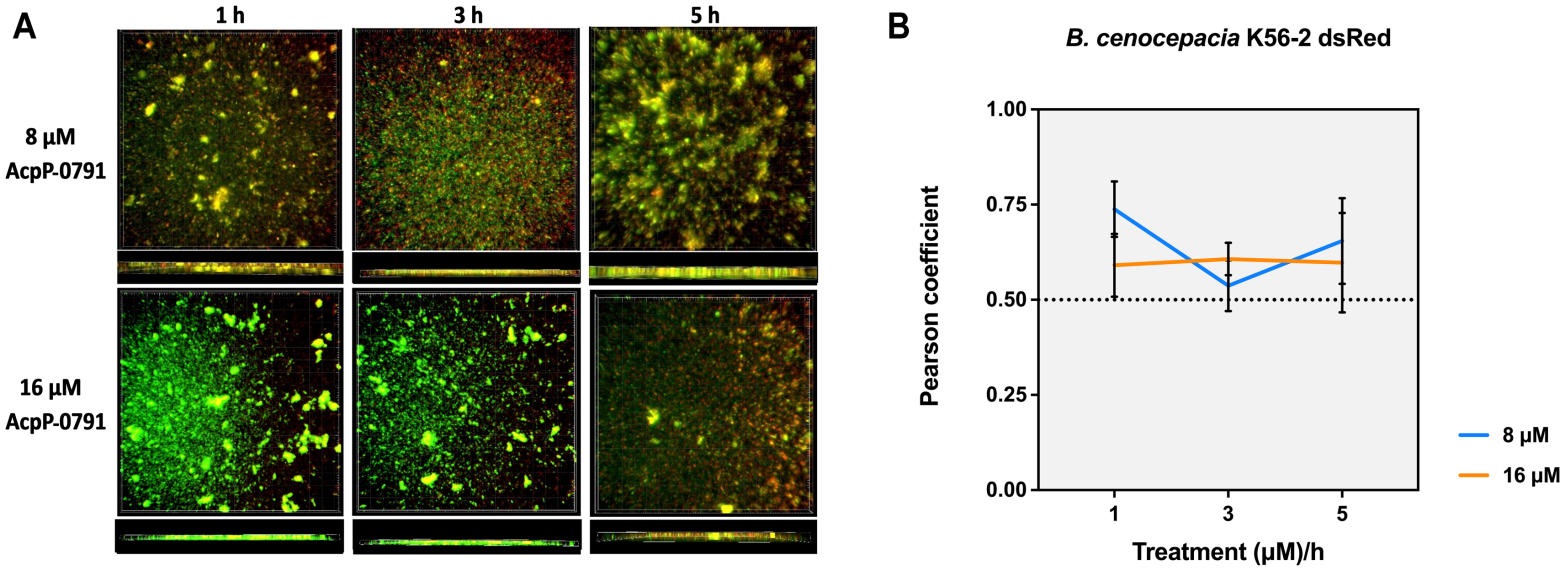
AcpP PPMOs co-localized with the Bcc. *B. cenocepacia* K56-2 dsRed biofilms (red) were grown for 72 h in a glass-bottomed 96-well plate with daily media changes. At 72 h, the biofilms were treated with a 0, 8, or 16 μM fluorescein-labeled AcpP-0791 PPMO (green) and incubated for 1, 3, and 5 h at 37°C. The treated biofilms were washed with buffered saline and fixed with 2.5% glutaraldehyde. **(A)** Top and side views of representative wells for each condition are illustrated using confocal microscopy. **(B)** Corresponding Pearson correlation coefficients are graphed.

At 72 h, the *B. cenocepacia* K56-2 DsRed biofilm (red channel) was treated with the fluorescein-labeled AcpP PPMO (Acp-0791; green channel) in a dose-dependent manner over time. Microscopic imaging was used to examine whether AcpP-0791 specifically co-localized with the bacteria in the biofilm. Overlap between the red and green channels was observed in both the 8- and 16 μM AcpP-0791 groups at all time points: 1-, 3-, and 5 h (Figure 5A).

This overlap was analyzed using Imaris, and a Pearson’s correlation coefficient was calculated to be >0.5 for each condition (Figure 5B). These findings indicate that the Bcc bacteria and AcpP PPMO co-localize with each other in the biofilm setting, providing additional evidence that the Bcc biofilm setting is not a deterrent to PPMO delivery.

### PPMOs were not toxic at bactericidal doses

**Figure 6:**
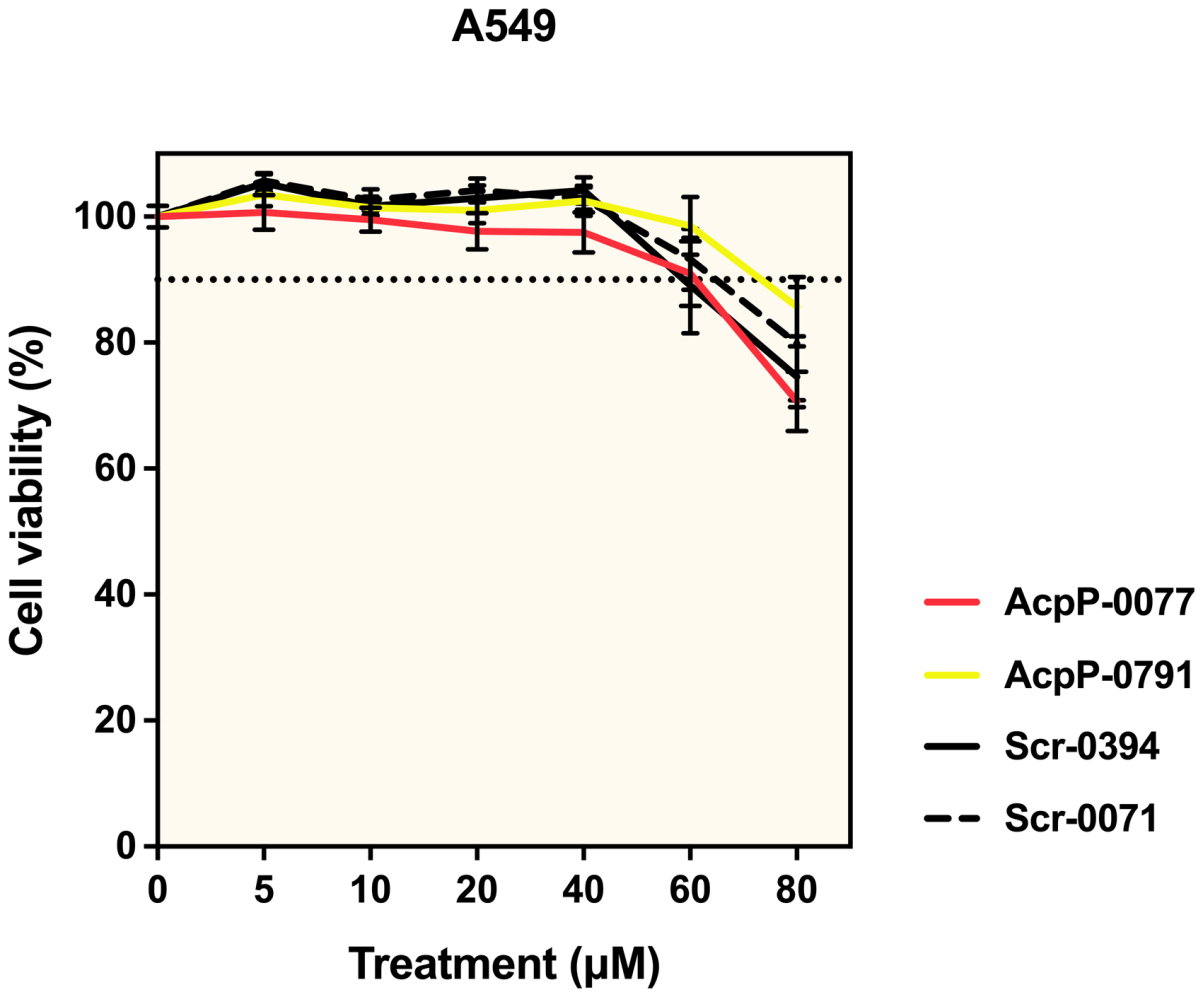
AcpP PPMO treatment is non-toxic to human type II pneumocytes (A549) at biofilm-reducing concentrations. Bactericidal concentrations (10–40 μM) of AcpP PPMOs do not reduce the viability of human alveolar epithelial cells. Cells were seeded into 96-well plates at 4000 cells/well and incubated at 37°C with 5% CO2 in DMEM/F12K (with glutamine, no phenol red, or antibiotics) to reach logarithmic growth. Then, the cells were treated with 5–80 μM AcpP PPMOs, and LDH release for each condition was quantified using the CyQUANT LDH Cytotoxicity Assay. Cytotoxicity was determined as per the manufacturer’s protocol, and relative cell viability was calculated (100% × 1 – cytotoxicity). Data are reported as mean percent viability (±SEM) and represent three independent experiments, each with three technical replicates. The dashed line represents 90% viability.

Previously, non-Bcc PPMOs have been shown to be well-tolerated in normal human bronchial epithelial cells (Ayhan et al., 2016; Geller et al., 2018). More recently, Bcc AcpP PPMOs tested in the CF lung cell line CFBE41o- exhibited low toxicity (Daly et al., 2018). However, AcpP-0077 and Scr-0394 used in the present study have different oligonucleotide sequences and peptide orientations. Additionally, given the higher doses required to treat a biofilm, we re-evaluated AcpP PPMO toxicity over a range of concentrations. The A549 cell line (immortalized type II pneumocyte) was used because it represents cells that could be affected by PPMO therapy in the context of lung infections. These cells were incubated with 5–80 μM PPMO for 24 h, and no toxicity was observed at concentrations up to 40 μM PPMO, as measured by LDH release (Figure 6). Viability was reduced by up to 10 and 30% at 60 μM and 80 μM, respectively. Notably, multi-log reductions (>3) were achieved in all Bcc strains tested at non-toxic doses, including the pan-resistant HI4277 strain with daily dosing (Figures 2B, 3).

## Discussion

The Bcc represents an important source of infection in individuals with cystic fibrosis (CF) and chronic granulomatous disease (CGD). Such infections pose a unique challenge due to the intrinsic antibiotic resistance present in these opportunistic pathogens, which has been observed in both clinical and environmental isolates (Mahenthiralingam et al., 2005; Sass et al., 2011; LiPuma et al., 2002). Despite this resilience, we previously demonstrated the successful application of peptide-conjugated phosphorodiamidate oligomers (PPMOs) targeting the well-conserved *Burkholderia* gene, *acpP*, *in vitro* and *in vivo* (Greenberg et al., 2010; Daly et al., 2018). Critically, this prior work showcased PPMO activity under conditions mimicking CGD, using a murine septicemia and neutrophil-killing model, and conditions resembling CF, using an artificial sputum and co-infection model.

However, the therapeutic challenge of treating Bcc infections is further complicated by its propensity to assemble biofilm (Conway et al., 2002; Hoiby et al., 2017; Lee et al., 2017). This adaptation has been correlated with persistent infection in the CF lung and has been shown to enhance resistance to antimicrobials compared to planktonic cells (Caraher et al., 2007; Desai et al., 1998; Traverse et al., 2013). Traditionally, the biofilm matrix is considered to confer antibiotic resistance by presenting structural and physiochemical barriers that limit diffusion and facilitate sequestration. Additional resistance mechanisms include the presence of persister cells and changes in gene expression in response to the unique microenvironment. These changes include a reduction in metabolic activity and the upregulation of efflux systems (Lebeaux et al., 2014; Buroni et al., 2014). Despite these defenses, PPMOs displayed potent antimicrobial activity in the biofilms of *Pseudomonas* and *Klebsiella*, offering a distinct therapeutic advantage over small-molecule antibiotics (Howard et al., 2017; Geller et al., 2018; Moustafa et al., 2021). Here, we show that the anti-biofilm activity of PPMOs is also retained in the Bcc. We assessed the activity of AcpP PPMOs on Bcc biofilms using multiple approaches, including actual quantification of viable bacterial burden, as well as measurements of overall biomass and metabolic activity. Microscopy qualitatively supported these findings, with SEM showing not just the destruction of intact bacteria but also a decrease in EPS.

It remains unclear why PPMOs retain activity in the biofilm setting across several medically important Gram-negative pathogens. This is in stark contrast with traditional small-molecule antibiotics, which have difficulty reaching bacteria shielded by this protective matrix (Messiaen et al., 2014; Conway et al., 2002; Caraher et al., 2007; Desai et al., 1998). We hypothesize that PPMOs, despite being high-molecular-weight compounds, derive their biofilm activity due to the peptide attachment as opposed to the PMO itself. The arginine-rich, positively charged residues facilitate delivery to the negatively charged membrane. In myocytes and cardiomyocytes, distinct endocytic pathways have been shown to mediate membrane translocation (Lehto et al., 2014). Interestingly, in one such pathway involving PPMO uptake via scavenger receptors, Ezzat et al. showed that PPMOs spontaneously assemble into micelles that retain a distinct size and charge (Ezzat et al., 2015). This observation is explained by the PPMO structure conferring amphipathicity, in which a relatively neutral PMO is conjugated to a highly positively charged peptide. Importantly, these findings also demonstrated that micelle self-assembly is a dynamic, reversible process. The physical disruption of the negatively charged biofilm environment with the positively charged antisense structure could be one mechanism that explains the antibiofilm activity of PPMOs despite their molecular weight.

PPMOs have several qualities that make them excellent candidates to be antimicrobial therapeutics, namely their stability, potency, high specificity, and target flexibility. However, it is essential to consider the potential of pathogens to develop resistance to PPMOs. Based on our current understanding, we hypothesize that potential resistance mechanisms may involve modulating the PPMO uptake or mutations in the target gene sequence. PPMO resistance in *Escherichia coli* was linked to mutations in *sbmA*, which encodes a peptide antibiotic active transporter (Puckett et al., 2012). These mutations conferred resistance to all PPMOs containing the C-N-N peptide moiety; however, mutants could be rescued by using alternative peptides. In this report, one antibiotic-resistant, epidemic strain, HI4277, showed decreased susceptibility to AcpP PPMO treatment at 24 h when compared to other strains. However, daily dosing of PPMOs resulted in a steady decline in biofilm burden, albeit at high concentrations. It remains unclear what makes this strain relatively resistant to PPMOs. Given its high level of resistance to other antibiotics and the progressive reduction in bacterial burden when treated with PPMOs over several days, the relative resistance is unlikely due to sequence-based target mutations and more likely related to entry. Future studies should focus on addressing this question in this strain and more broadly across the Bcc. Although we have previously demonstrated that PPMOs remain active in synthetic sputum, *in vivo* toxicity studies are still needed to determine whether PPMOs have a viable therapeutic index of activity in specific clinical settings, such as the treatment of pulmonary Bcc infections. This includes determining efficacy and toxicity with targeted direct-to-the-lung therapy. Despite these uncertainties, the ability of PPMOs to retain activity in Bcc biofilms is a promising advantage of this emerging antibacterial approach.

## Data Availability

The raw data supporting the conclusions of this article will be made available by the authors, without undue reservation.
