# Supplementary material for: Antisense phosphorodiamidate morpholino oligomers retain activity in Burkholderia cepacia complex biofilm
Source: Front Microbiol. 2025 Sep 23;16:1660799. doi: 10.3389/fmicb.2025.1660799 (PMC12500672; doi:10.3389/fmicb.2025.1660799)
Supplement: Supplementary file 1 [file Supplementary_file_1.pdf]

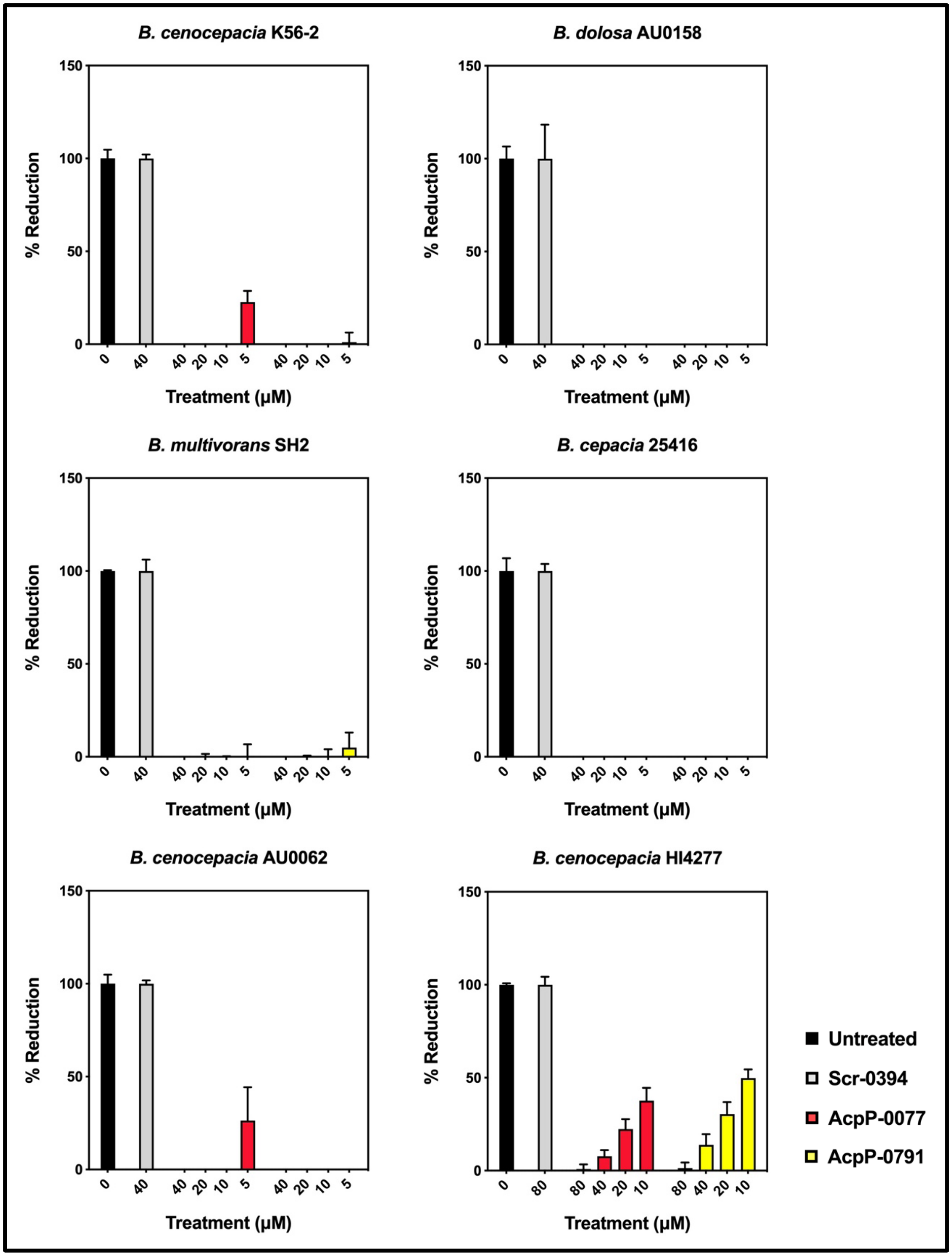

Supplemental FIG 1. Metabolic activity of PPMO-treated biofilm. Bcc biofilms were grown and treated as described in FIG 2. Samples were dosed with AcpP-0077 (red), AcpP-0791 (yellow), or scramble sequence control (gray). Metabolic activity was assessed by resazurin assay. Mean (± SEM) reduction in activity from 100% (dashed line) is depicted. Data represent three assays with three technical replicates each.

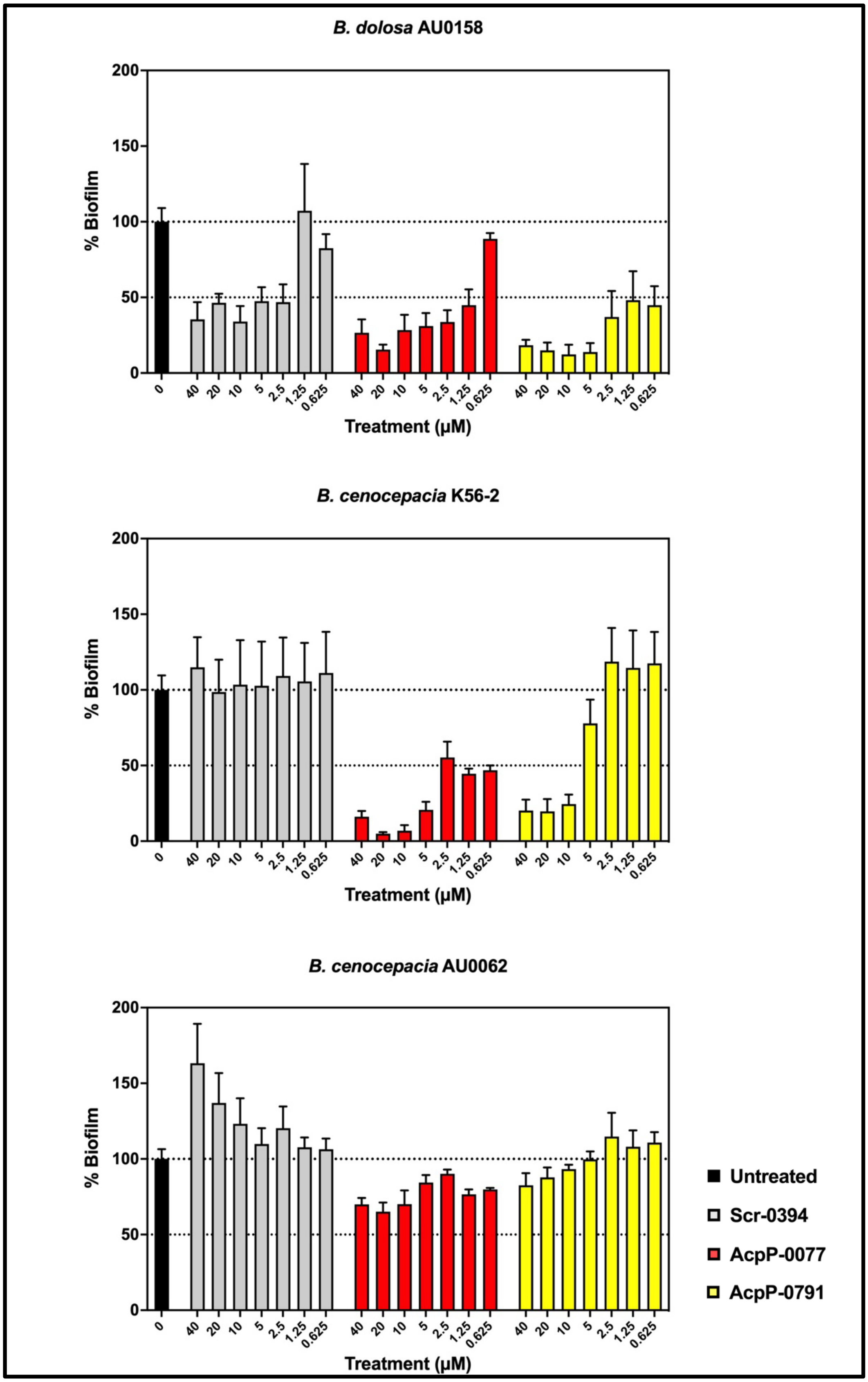

Supplemental FIG 2. Total biofilm burden of PPMO-treated biofilm. Bcc biofilms were grown and treated as described in FIG 2. Samples were dosed with AcpP-0077 (red), AcpP-0791 (yellow), or scramble sequence control (gray). Biofilm burden (bacteria and exopolysaccharides) was assessed by crystal violet assay. Mean (± SEM) percentage of remaining biofilm is given, after normalization to an untreated control. Data represent 1–2 assays per condition, with three technical replicates each.

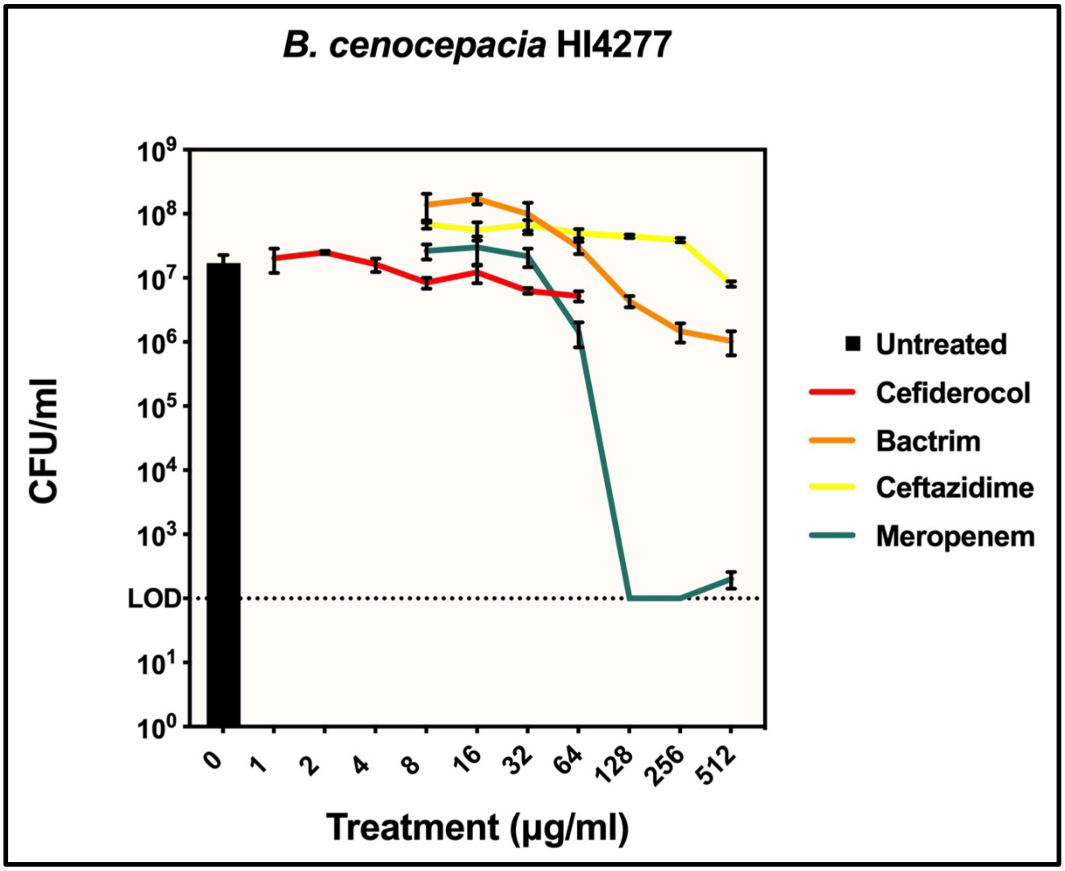

Supplemental FIG 3. HI4277 demonstrated resistance to multiple classes of antibiotics, even at high doses. Biofilms were grown, treated, and quantified as described in FIG 2. Samples were dosed with various classes of antibiotics at 1–512 µM. An untreated control was included. Data represent one independent experiment, with three technical replicates each. Limit of detection is indicated by the dashed line.
